# The Effect of “Online–Simulation–Bedside” Three-Step Teaching Method in Team Cardiopulmonary Resuscitation Skills Training of Emergency Department and Critical Care Nursing Interns—An Analysis Based on Kirkpatrick Model

**DOI:** 10.1155/jonm/8624274

**Published:** 2025-02-24

**Authors:** Huan Liu, Hui Huang, Miaoya Li, Ping Mao, Aidi Zhang, Yanting Sun, Zhaoxun Liu, Hong Tao, Sha Zhao, Yuting Xia, Jiandang Zhou, Jinxin Liu

**Affiliations:** ^1^Nursing Department, The Third Xiangya Hospital, Central South University, Changsha, Hunan, China; ^2^Xiangya Nursing School, Central South University, Changsha, Hunan, China; ^3^Laboratory Department, The Third Xiangya Hospital, Central South University, Changsha, Hunan, China; ^4^Foreign Studies College, Hunan Normal University, Changsha, Hunan, China

**Keywords:** Kirkpatrick model, nursing interns, simulation training, team cardiopulmonary resuscitation

## Abstract

**Aim:** This study designed a three-step teaching method of “online–simulation–bedside,” which was applied to the teaching of cardiopulmonary resuscitation skills in the team of emergency and critical care nursing interns, and the effectiveness of this teaching method was evaluated using the Kirkpatrick model.

**Background:** Mastering knowledge of cardiopulmonary resuscitation and emergency techniques is necessary for nursing interns to fulfill their roles. At present, the first aid awareness and rescue skills of nursing interns are not optimistic. Training programs can improve the cardiopulmonary resuscitation ability of nursing students, but most of them only evaluate the changes in cardiopulmonary resuscitation ability before and after training and rarely use educational evaluation theories and models to evaluate the effectiveness of cardiopulmonary resuscitation training research.

**Methods:** This is a quasi-experimental research design: pretest and posttest design. Our research focuses on nursing interns who interned in the emergency and critical care departments of a hospital from September 2023 to January 2024. Adopting the three-step teaching method of “online-simulation-bedside”, provide team cardiopulmonary resuscitation skills training for emergency and critical care nursing interns. The four levels of Kirkpatrick's model, that is, reaction, learning, behavior, and result, were applied for the evaluation together with questionnaires. Conduct a baseline survey before online learning. Evaluate team working ability, clinical thinking ability, confidence in skills, and active participation on the first day after simulated training and the 30th day of clinical practice. On the 30th day of clinical practice, satisfaction and skill level assessments will also be conducted.

**Results:** At the reaction level, the satisfaction rate of nursing interns is 98.80%. At the learning level, there was a statistically significant difference (*p* < 0.001) in the team work ability and clinical thinking ability of nursing interns before training, on the first day after training, and on the 30th day of clinical practice. At the behavioral level, there was a statistically significant difference (*p* < 0.001) in the skill confidence and participation initiative of nursing interns before training, on the first day after training, and on the 30th day of clinical practice. At the result level, on the 30th day of clinical practice, the nursing intern team's cardiopulmonary resuscitation skills assessment scores were all good or above, with an average score of 90.09 ± 1.58.

**Conclusion:** The Kirkpatrick model can be used to evaluate the effectiveness of cardiopulmonary resuscitation skills training for nursing intern teams. Nursing interns are highly satisfied with the “online–simulation–bedside” three-step teaching method, which can improve their teamwork ability, clinical thinking ability, active participation, and skill confidence. In addition, through training, students can effectively apply the learned content to clinical practice and possess a good level of skills.

**Implications for Nursing Management:** Managers can develop a distinctive team cardiopulmonary resuscitation training model based on this, improve the team cardiopulmonary resuscitation skills of nursing interns, and cultivate more nursing talents with high-level emergency capabilities for hospitals.

## 1. Introduction

Cardiac arrest is an important global public health problem. 500,000 people die of cardiac arrest in the United States every year [1], and 544,000 people suffer from sudden cardiac death in China every year [2]. Cardiopulmonary resuscitation (CPR) is a therapeutic method to rescue cardiac arrest, and early implementation of CPR is the most effective rescue measure [3]. Most cardiac and respiratory arrests occurring in hospitals are first recognized by nurses. Nurses play a very important role in the rescue of critically ill patients. Nurses' team cooperation ability and professional and technical level have a far-reaching impact on the success rate of patients' rescue [4]. Nursing interns are the reserve force of the future nursing career. It is necessary for them to master the measures of CPR and first aid techniques. It should be decided and used uniformly as just the basic life support. However, the first aid team awareness and rescue skills of nursing interns are not optimistic [5]. In particular, Emergency, Intensive Care Unit (ICU), and other special clinical medicine disciplines have a heavy workload, a fast pace, high tension, and many CPR rescue events. Nursing interns often have fear in the face of CPR rescue due to an unfamiliar clinical environment, a lack of experience, and other reasons [6]. The negative emotions and pressure brought by CPR have seriously affected the professional and personal lives of nursing interns and even affected their future career choices [7]. It is of great significance to carry out team CPR skills training for nursing interns in acute and critical departments.

At present, nursing intern skills teaching mainly adopts bedside teaching, which is a patient-centered clinical teaching method with teachers leading students to conduct on-site teaching and has been widely used in undergraduate clinical teaching in medical colleges [8]. In recent years, the limitations of bedside teaching have become increasingly prominent. Theoretical courses are seriously disconnected from bedside teaching, and there is a lack of an effective transition mechanism, resulting in the poor effect of bedside teaching [9]. Most of the patients admitted to emergency and critical care departments are critically ill patients, and most of the nurses carry out team CPR on real patients in emergency and sudden situations. The stressful environment is not suitable for nursing interns to learn CPR skills, and team CPR performed by unskilled students may put patients at risk. At this stage, a single teaching mode can no longer meet the needs of teaching, so it is necessary to explore the joint teaching mode to realize the effective transition of CPR skills training for nursing interns from theory to bedside teaching.

Simulation teaching is a method of clinical teaching and practice using simulation technology to create high-fidelity molds or simulated clinical scenarios instead of real patients [10]. Trainees are trained in simulation scenarios of communication skills, leadership, and teamwork before contacting real patients, which can enhance their confidence in their medical abilities and skills, thereby reducing medical errors to a large extent, improving patient safety, and significantly reducing healthcare costs in the long run [11]. The traditional “apprenticeship” learning in nursing education is gradually changing to simulation-based “experiential” learning, and simulation teaching has gradually become a new development trend in nursing education [12]. Studies have shown that simulation-based CPR training can improve the professionalism of nursing students in terms of knowledge, performance, and self-efficacy and is regarded by students as an interesting and effective teaching mode [13]. The use of high-fidelity simulators contributes to the development of clinical reasoning, critical thinking, problem solving, decision making, and clinical judgment in nursing students [14]. In addition, with the rapid development of network technology, online learning has been widely used in higher education [15], and online learning is defined as “an educational method that facilitates learning through the application of information technology and communication, providing learners with access to all required educational programs” [16]. Online learning gives students better access to learning resources that can be accessed regardless of time and geographic location [15].

Therefore, this study constructed a three-step teaching model of “online–simulation–bedside” to provide team CPR skills training for emergency and critical care nursing interns. First, students learn relevant theoretical knowledge online, then conduct simulated teaching, and finally enter clinical practice learning. Related studies have shown that simulation-based CPR training can improve the professional level of nursing students in terms of knowledge, performance, and self-efficacy and is regarded by students as an interesting and effective teaching model [13]. The use of high-fidelity simulators can help cultivate the clinical reasoning ability, critical thinking ability, problem-solving ability, decision-making ability, and clinical judgment ability of nursing students [14]. Although relevant studies have found that training can improve the CPR ability of nursing students, they have only evaluated the changes in CPR ability before and after training [17–19] and there is currently no research using educational evaluation theory and models to evaluate the effectiveness of CPR training.

Evaluating the effectiveness of training models is a crucial step in the training process. This requires a systematic evaluation model. Kirkpatrick's four-level model of evaluation is a mature teaching evaluation tool that divides training effectiveness into four levels: reaction, learning, behavior, and result [20]. It has been widely used in medical training and education fields [21]. The Kirkpatrick model was used to evaluate the effectiveness of virtual reality (VR) intravenous injection training programs for nurses and nursing students [22]. The Kirkpatrick model evaluates the effectiveness of clinical mental health education for nursing students [23]. Evaluation of shift handover training plan for nursing students based on Kirkpatrick model [24].

This study constructed a three-step teaching model of “online–simulation–bedside” to provide team CPR skills training for emergency and critical care nursing interns. Then, the effectiveness of the training model was verified using the Kirkpatrick model, and the satisfaction of students with the training model was verified. In addition, it also aims to verify the effectiveness of this teaching model in improving the teamwork, clinical thinking ability, skill confidence, and active participation of nursing interns. At the same time, we also innovatively explored the effect of nursing interns transferring training content to clinical practice and tested the team CPR skills of nursing interns after clinical practice. The results of this study can provide theoretical basis for the construction of CPR training mode for nursing interns and the decision making of managers.

## 2. Methods

### 2.1. Design

This was a single group, pretest–posttest study. This study is based on Kirkpatrick's four-level model to evaluate the training effect of “online–simulation–bedside” three-step teaching method on CPR skills of critical care interns.

### 2.2. Participants and Settings

Participants were nursing students who were interns from September 2023 to January 2024 in the Department of Emergency and Critical Care of a hospital. Inclusion criteria were as follows: enrolled in a full-time medical school nursing program; aware of and voluntarily participating in the entirety of this study; and signing an informed consent form; exclusion criteria were as follows: having a team CPR simulation teaching learning experience and participating in another teaching study. Of the 103 students who met the inclusion criteria, 17 students decided to voluntarily leave the study for different reasons, and 86 students who completed the simulation training and questionnaire were included in the final analysis.

### 2.3. Intervention

**Figure 1:**
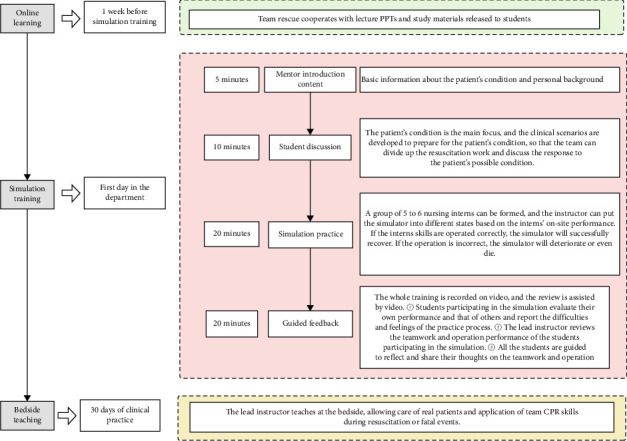
Team CPR teaching process.

Simulation teaching setup and preparation: The location of each training session was set up in the simulation classroom of the hospital's Clinical Skills Center. Before each training session, all items and medicines are prepared in accordance with work routines. In addition, video equipment is equipped to record the whole training. Using a high-simulation simulator to simulate cardiac arrest, the simulation teacher will design the vital signs nodes and corresponding changes in data needed for the case condition in advance. During the simulation process, the instructor can let the simulator enter into different preset scenarios or states according to the trainee's on-site treatment and show them in real time through the signs or simulation monitors. Nursing trainees from the Department of Emergency and Critical Care Medicine are randomly grouped for simulation training in monthly rotations. The details are introduced in Figure 1.

### 2.4. Study Instruments

This study designed its own evaluation tool, by the team members and five simulation teaching experts, after a joint discussion to form the evaluation tool of the satisfaction of training (reaction level), teamwork ability (learning level), clinical thinking ability (learning level), skill confidence (behavior level), and active participation (behavior level), all of which were scored using 5-point Likert scale, with a minimum of 1 point and a maximum of 5 points. In addition, the skills test scores (result level) were evaluated using the Team CPR Assessment Score Sheet designed in the previous study, which was divided into four dimensions: preparation of supplies, resuscitation process, operational skills, and team cooperation for evaluation, with a maximum of 100 points and a minimum of 0 points, > 90 points as excellent, 85–90 points as good, 80–84 points as qualified, and less than 80 points as unqualified. The score sheet was developed by the nursing team of the emergency department of our hospital in 2019 and has been widely used in the clinical assessment of CPR for our team, with a good evaluation effect.

### 2.5. Data Collection

Data were collected in the longitudinal setting of three-wave measurements (i.e., T0 = baseline measurement collection intervention, T1 = day 1 after the second measurement collection intervention, and T2 = day 30 after the third measurement collection intervention). During the questionnaire survey, the research subjects are required to fill in the questionnaire independently according to their own actual situation to ensure the reliability of the information. In the event of some difficult-to-understand entries, the researcher will explain them accordingly, and a unified guideline will be adopted. Medical simulation teaching experts were consulted for advice on the teaching design to ensure the feasibility and clinical significance of the study. The members of the research team have received training and knowledge related to simulation teaching.

### 2.6. Statistical Analysis

SPSS 23.0 software was used to analyze the data. Means, standard deviations, frequencies, and percentages were used to describe the demographics of the students. Means, standard deviation descriptions, and percentages were used to describe nursing interns' satisfaction, teamwork ability, clinical thinking ability, skill confidence, active participation, and test scores on day 1 after simulation training and day 30 of clinical practice. ANOVA was used to analyze whether there was any difference between pre- and post-training. The *t*-test was used to analyze the satisfaction, teamwork ability, clinical thinking ability, skill confidence, active participation, and skill test scores of nursing interns who experienced real patient CPR or not. *p*=0.05 was used as the test standard.

### 2.7. Ethical Considerations

This study was approved by the Review Committee of the Third Xiangya Hospital of Central South University (Approval No: Fast 23945). Informed consent was obtained from the patients before data collection, and the information of the students was kept properly. This study protected the personal privacy of nursing students.

## 3. Results

### 3.1. Participant Characteristics

The age range of 86 nursing interns was 19–24 years with a mean age of 20.99 ± 0.94, 8 (9.30%) were males, and 78 (90.70%) were females. They had practiced for 4–9 months before the training, with an average internship duration of 6.55 ± 1.07 months.

### 3.2. Outcome Measures

#### 3.2.1. Nursing Interns' Satisfaction (Reaction Level)

The score of nursing interns' satisfaction with the training was 4.57 ± 0.52, 40.70% of the students were satisfied with the training mode, and 58.10% of the students were very satisfied with the training mode.

#### 3.2.2. Impact of Training on Nursing Interns' Teamwork Ability and Clinical Thinking Ability (Learning Level)

**Figure 2:**
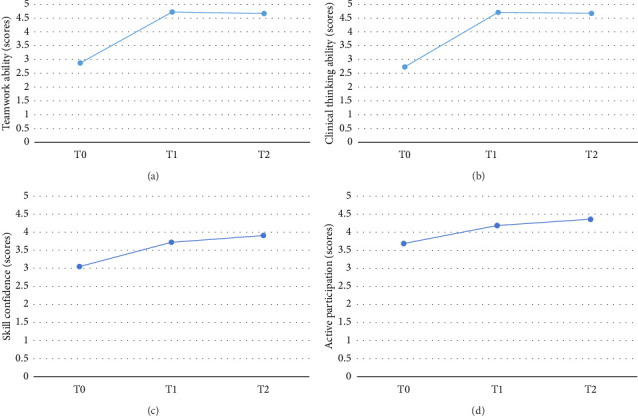
Mean changes of learning level and behavioral level. (a) Teamwork ability, (b) clinical thinking ability, (c) skill confidence, and (d) active participation.

On the first day after simulation training and the 30th day of clinical practice, compared with before training, students' teamwork ability and clinical thinking ability significantly improved.  Table 1 and Figure 2 show this in detail.

#### 3.2.3. The Effect of Training on Confidence and Active Participation in Team CPR Skills of Nursing Interns (Behavioral Level)

On the first day after the simulated training and the 30th day of clinical practice, compared with before the training, the confidence and active participation of the student team in CPR skills significantly improved; Table 1 and Figure 2 show this in detail.

#### 3.2.4. The Test Score of Team CPR Skills of Nursing Interns (Result Level)

All nursing interns' team CPR skills assessment scores were good or above with the highest score of 92.00 and the lowest score of 85.5 with a mean score of 90.09 ± 1.58.

#### 3.2.5. Analysis of the Impact of Experiencing Real CPR Events in Patients on the Effectiveness of Training

Nursing interns who experienced patient-authentic CPR had significantly higher skill confidence (behavior level) and skill levels (outcome level) than students who did not, *p* < 0.05. The details are introduced in Table 2.

## 4. Discussion

In this study, we developed a three-step teaching method of “online–simulation–bedside” for the training of CPR skills for nursing internship teams. Then, we used the Kirkpatrick model to validate the effectiveness of the training model, verify students' satisfaction with the teaching mode, and verify the effect of the mode on improving teamwork and clinical thinking ability, skill confidence, and active participation. In addition, we also tested the students' CPR skills level after the training.

With regard to the reaction level, the results of this study show that students are highly satisfied with this teaching mode. The possible reason is that “online teaching” can present knowledge in detail, and “simulated teaching” as an experiential educational tool is a means of connecting theory and practice, which can narrow the gap between theory and practice [25]. The three-step teaching model with simulation as a bridge increases the fun, participation, and interaction of learning for nursing interns and is a good learning experience. Students believe that simulation is an interesting and useful teaching method [19].

With regard to the learning level, this study found that on the first day after simulated training and the 30th day of clinical practice, students' teamwork ability and clinical thinking ability were improved compared to before training. Rupert also confirmed this point, stating that after receiving simulation-based CPR training, nurses' CPR skills and teamwork awareness improved by 10% [26]. In addition, the “review” process in the simulation can stimulate students to actively think, discover problems, and transform passive knowledge acceptance into active knowledge acquisition, thereby generating positive cognitive outcomes [27]. Simulated training creates a safe and threat-free environment, allowing learners to improve their leadership skills, critical thinking, decision making, problem solving, and prioritization abilities [19], which has a positive impact on thinking skills and lifelong learning [28].

With regard to the behavior level, on the first day after simulated training and the 30th day of clinical practice, compared with before training, the confidence and active participation of the student team in CPR skills significantly improved. The possible reason is that students can practice repeatedly through simulation training, giving them more opportunities to reflect on themselves and repeat learning until they have confidence in their CPR skills and abilities [29]. In addition, the “review” stage is a reflection process conducted after clinical simulation training [30], where teachers and learners interact to jointly reflect on the thinking and behavior of learners during the learning process. Reviewing helps to transform experience into knowledge through examination, reflection, and reassessment of the scene [31]. When participants' behavior is positively reinforced and guided to improve, they will have more confidence in their clinical skills. Simulated courses have improved the professional knowledge, clinical practice ability, and learning confidence of nursing interns, preparing them for real-world clinical practice [32], reducing anxiety in clinical practice [33], and thereby enhancing their participation initiative. Simulation training, as a supplement to clinical practice, significantly improves students' satisfaction, confidence [34], and learning outcomes [35].

With regard to the result level, all nursing intern teams achieved good or above scores in the assessment of CPR skills. The possible reason is that simulation provides the closest learning experience to reality [36], and students have the opportunity to detect and correct their mistakes through repeated practice during the simulation process, which helps improve their clinical professional skills [37]. Reviewing is a fundamental element of learning, and through continuous reflection, learning experiences can be transformed into practice [38]. Simulated teaching is a bridge for students to transition from the learning environment to clinical practice and has advantages in improving nursing students' professional skills [39]. Through the process of “online,” “simulation,” and then “bedside,” intern nursing students gradually deepen their understanding of team CPR skills, forming a process from “recognition,” “understanding” to “application,” completing the transformation from theory to practice, and enabling nursing students to achieve good operational skills.

This study also found that nursing interns who experienced real CPR in clinical practice had significantly higher skill confidence (behavioral level) and skill level (result level) than students who did not experience it. Although simulation is beneficial, nursing interns must practice clinical skills in a real clinical learning environment to integrate theoretical and practical knowledge, skills, and abilities [40]. Clinical practice is crucial for intern nursing students, as they can transform theoretical knowledge into personal clinical practice abilities through clinical practice [41].

The results of this study showed that on the 30th day of clinical practice, although the scores of teamwork ability and clinical thinking ability were significantly higher than before training, they were slightly lower than on the 1st day after training. The long-term effect of this teaching model on students' learning needs to be strengthened, and the retention of training effectiveness has declined over time. In a cross-sectional study, 205 medical students who received CPR training had an average retention rate of 90% after one month, 74% after 18 months, 62% after 30 months, and 61% after 42 months [42]. Spooner also found that CPR skills significantly decreased after 6 weeks of training [43]. A study by Araujo showed that after 6 months of training, theoretical and practical knowledge of CPR decreased by 14.5% [44], and skills declined faster than knowledge [45]. Retraining is considered an effective method to prevent the loss of acquired abilities [46]. Reviewing or strengthening courses has been proven to effectively improve the retention rate of CPR skills [47, 48]. However, the optimal timing for repetition is still unclear. According to the skill retention prediction curve, in order to maintain at least 70% of skills, some studies suggest using 18–24 months as the shortest retraining interval [42]. Al and Al also found that retraining 6 months after initial CPR training can promote skill retention for 12 months [49]. There are also studies indicating that the ideal retraining period is 3–6 months [46, 50]. Anderson et al. proposed that monthly interval training is more effective in improving skill retention rates than training every 3, 6, or 12 months [51]. Therefore, in order to promote the retention of CPR skills, nursing managers can regularly conduct team CPR retraining for nursing interns.

## 5. Limitation

Regarding the limitations of this study, firstly, we only used comparisons before and after training. The next step will be to establish a set of routine training as a control group to control for confounding factors. In addition, the participants are from a teaching hospital. This feature may limit the generalizability of the results of this study. The next step will be to conduct multicenter research, apply the designed training model to other institutions, verify its effectiveness, and explore teaching models suitable for nationwide dissemination.

## 6. Conclusion

In this study, we used the Kirkpatrick model as a training assessment tool to comprehensively evaluate the effectiveness of the “online–simulation–bedside” three-step teaching approach. Research shows that the teaching approach is an effective way to improve CPR skills in acute critical care trainee teams at the reaction, learning, behavior, and result levels. This teaching model was recognized by nursing interns, which improved nurses' ability in teamwork, clinical thinking, active participation, and skill confidence. This lays the foundation for the construction of clinical nursing training and evaluation methods.

## 7. Implications for Nursing Management

This study provides important management implications for the training and effectiveness assessment of care managers. Nurse managers should realize that the three-step “online–simulation–bedside” teaching approach can improve the effectiveness of nursing intern teams on CPR skills. Therefore, nursing management should develop effective measures to apply this model in CPR teaching for nursing interns. It is recommended that nurse managers use the Kirkpatrick model as an evaluation tool to evaluate the effectiveness of team CPR skills training among nursing interns.

## Figures and Tables

**Table 1:** Comparison of nursing interns' teamwork ability, clinical thinking ability, skill confidence, and active participation scores in team CPR skills at different time points.

Outcomes	Time point	Score Mean ± SD	*F*	*p*
Teamwork ability	T0	2.87 ± 0.48	335.93	< 0.001
	T1	4.72 ± 0.52		
	T2	4.66 ± 0.59		

Clinical thinking ability	T0	2.73 ± 0.45	428.759	< 0.001
	T1	4.71 ± 0.53		
	T2	4.67 ± 0.54		

Skill confidence	T0	3.04 ± 0.77	41.48	< 0.001
	T1	3.72 ± 0.64		
	T2	3.91 ± 0.52		

Active participation	T0	3.69 ± 0.86	20.46	< 0.001
	T1	4.19 ± 0.54		
	T2	4.36 ± 0.71		

*Note:* T0: pretraining; T1: day 1 after simulation training; T2: day 30 of clinical practice. F = analysis of variance (ANOVA).

**Table 2:** Comparison of outcome indicators between nursing interns who experienced and did not experience patient CPR.

Outcomes	Group	Numbers	Scores	*t*	*p*
Satisfaction	Inexperienced	51	4.56 ± 0.53	0.413	0.681
	Experienced	35	4.57 ± 0.50		

Teamwork ability	Inexperienced	51	4.66 ± 0.58	0.074	0.942
	Experienced	35	4.65 ± 0.59		

Clinical thinking ability	Inexperienced	51	4.64 ± 0.59	−0.564	0.574
	Experienced	35	4.71 ± 0.45		

Active participation	Inexperienced	51	4.25 ± 0.74	−1.663	0.100
	Experienced	35	4.51 ± 0.65		

Skill confidence	Inexperienced	51	3.80 ± 0.60	−2.258	0.027
	Experienced	35	4.05 ± 0.33		

Skill test score	Inexperienced	51	89.78 ± 1.92	−2.273	0.026
	Experienced	35	90.55 ± 0.68		

## Data Availability

The data that support the findings of this study are available on request from the corresponding authors. The data are not publicly available due to privacy or ethical restrictions.
